# Association of *PTPN22* rs2476601 Polymorphism with Rheumatoid Arthritis and Celiac Disease in Khuzestan Province, Southwestern Iran

**DOI:** 10.6091/.21.1.61

**Published:** 2017-01

**Authors:** Zahra Abbasi, Seyed Reza Kazemi Nezhad, Mahdi Pourmahdi-Broojeni, Elham Rajaei

**Affiliations:** 1 Department of Genetics, Faculty of Science, Shahid Chamran University of Ahvaz, Ahvaz, Iran; 2 Department of Food Hygiene, Faculty of Veterinary Medicine, Shahid Chamran University of Ahvaz, Iran; 3 Department of Internal Medicine, School of Medicine, Ahvaz Jundishapur University of Medical Sciences, Ahvaz, Iran

**Keywords:** Celiac disease, PTPN22, Rheumatoid factor, Rheumatoid arthritis

## Abstract

**Background:**

Single-nucleotide polymorphism (SNP) rs2476601 within protein tyrosine phosphatase non-receptor type 22 gene (*PTPN22*) has been shown to be a risk factor for different autoimmune diseases. This study explored the association of 1858 C/T SNP with rheumatoid arthritis (RA) and celiac disease (CD) in a region covering south-west of Iran.

**Methods:**

Totally, 52 patients with CD, 120 patients with RA, and 120 healthy subjects were selected. The samples were genotyped for the rs2476601 in *PTPN22* gene using the tetra-amplification refractory mutation system polymerase chain reaction.

**Results:**

The frequency of +1858T risk allele was significantly increased in both RA (*P*=0.021, OR=2.56, 95%CI=1.19-5.47) and CD (*P*=0.002, OR=3.87, 95%CI=1.68-8.95) patients, as compared to the control group. However, no association was found between the +1858C/T *PTPN22* gene SNP and the anti-cyclic citrullinated peptide and rheumatoid factor positivity in RA patients.

**Conclusions:**

*PTPN22* gene could play a crucial role in people’s susceptibility to certain autoimmune diseases.

## INTRODUCTION

Autoimmune diseases are chronic conditions distinguished by the loss of immunological tolerance to self-antigens[1] and affect 5–7% of the population of the world[^2^]. Rheumatoid arthritis (RA) is characterized by synovial inflammation, hyperplasia, cartilage and bone destruction, auto-antibody production (rheumatoid factor [RF]), anti-cyclic citrullinated peptide (CCP) and the decreased quality of life[3]. Its prevalence is approximately 0.5-1% worldwide and afflicts people of all races. The incidence of RA in women is three times higher than in men[4]. RA shares a number of pathogenic mechanisms with other autoimmune disorders. One of the autoimmune diseases found to have pathogenic mechanisms comparable to those seen in RA is the celiac disease (CD). It is a chronic intestinal inflammatory disorder developed through intolerance to gluten, a major dietary protein in wheat, and related proteins from barley and rye[5]. The prevalence of CD has been estimated to be roughly 0.5%-1% in different parts of the world[6]. The disease can clinically appear at any age but often occurs in the first few years of life. Autoimmune disorders such as RA and CD have a complex genetic background. A family-based epidemiological study has suggested that there is a shared genetic basis between the two autoimmune diseases[7].

Among the genetic factors, the most important genes are major histocompatibility complex genes, which create susceptibility to RA and CD. The second group of genes includes non-major histocompatibility complex. Among this second group, the protein tyrosine phosphatase non-receptor type 22 gene (*PTPN22*) is considered as the most important member[8]. *PTPN22* gene is located in chromosome 1p13 and encodes the lymphoid protein tyrosine phosphatase (LYP), which plays a prominent role in moderating signaling through the T-cell receptor[9] and negative control of T-cell activation in T-cell development in later stages[10].

There are various Single-nucleotide polymorphisms (SNP) of *PTPN22* gene affecting many autoimmune diseases but do not carry the same risk. The most common SNP of *PTPN22* gene is rs2476601 (1858C/T)[11]. A gain function mutation in this SNP at position 1858 in *PTPN22* gene transforms cytosine to thymine, which affects amino acid 620, an arginine to tryptophan missense polymorphism that alters the protein function[12].

*PTPN22* C1858T belongs to a growing family of shared autoimmunity loci, which are associated with various autoimmune disorders. The +T1858 allele increases the risk of developing RA and lupus[13]. At the same time, this allele might be a source of protection against Crohn’s disease and have no effect on multiple sclerosis[14-16]. However, data gleaned from different populations are very different, and some studies have demonstrated that there is no association between 1858C/T SNP and autoimmune diseases[16,17]. In RA disease, *PTPN22* R620W gene polymorphism reveals a wide variation in allele frequency between different populations, whereas it is absent in some other populations, especially Asians. In addition, more data are required to prove the existence of an association with CD[17].

Santin *et al*.[18] have demonstrated a relationship between 1858C/T SNP and CD disease, whereas Rueda *et al*.[17] have found no association between *PTPN22* C1858T polymorphism and CD. Considering the existence of discrepancies between the available data, the aim of this study was to analyze the association of +1858C/T *PTPN22* polymorphism with RA and CD in a population in the southwest of Iran.

## MATERIALS AND METHODS

### Subjects

In this study, all the patients and the control group were chosen from Khuzestan Province in the southwestern region of Iran. The study was approved by the ethics committee of Jundishapur University of Medical Sciences and carried out after the procurement of a written consent from all the subjects.

### Patients

#### Rheumatoid arthritis

In total, 120 RA patients, 96 women and 24 men, with an age range of 16–75 years were classified according to American College of Rheumatology 2010 criteria[19]. All the subjects were patients of the Rheumatology Clinic at Golestan Hospital in Ahvaz, Iran. RF 20 IU/mL was considered as RF positive. The presence of anti-CCP antibodies was determined, and a cut-off point of >5 U/mL was used as a stringent criterion for anti-CCP positivity.

#### Celiac disease

The study population covered 52 CD patients with clinical symptoms of CD, which diagnosed based on the European Society for Pediatric Gastroenterology, Hepatology and Nutrition criteria[20]. A total of 52 subjects, 36 women and 16 men with an age range of 9–51 years participated in the study. All the subjects were examined at a gastroenterology clinic in Razi Hospital of Ahvaz and by members of the Iranian Society of celiac disease in Khuzestan Province, Iran.

### Control group

In total, 120 healthy subjects, including 97 women, and 23 men with an age range of 26–76, were selected from Golestan Hospital. None of the subjects had a family history of autoimmune diseases.

### Genotyping of the +1858C/T PTPN22 gene polymorphism

The genomic DNA of the patients and the members of the control group were obtained from peripheral blood leukocytes using the Salting-out method[21]. The genotyping of the +1858C/T *PTPN22* gene SNP (rs2476601) was performed using the tetra-amplification refractory mutation system polymerase chain reaction technique, which was designed for the detection of *PTPN22* R620W (rs2476601) polymorphism[21]. Besides, the following outer and inner primers were used: forward outer: 5’-ATTAA CCACCAATCCAACATCCAGAC-3’ and reverse outer: 5’-CTTTCCCTCCACTGAGGACAAAGTT-3’ and forward inner: 5’-TCTCTTTCACTTCCTACAGA TGCTCA-3’ and reverse inner: 5’- AGCCTTCAAGA CCTGGCGCA-3’). The 25-µL PCR reactions contained 1 µL template DNA (~100 ng µL^−1^), 1 µL of each primer (10 µM), 12.5 µL PCR Premix and 7.5 µL sterile distilled water. Thermal cycling was achieved through the initial denaturation step at 95°C for 5 min, 35 cycles of denaturation at 94°C for 30 s, annealing at 60°C for 1 min, extension at 72°C for 1 min and a final extension at 72°C for 5 min. The size of PCR product was 213 bp for the C allele and 151 bp for the T allele, whereas the size of products amplified by the two outer primers was 314 bp. All products were analyzed on a 2.5% agarose gel. The genotypes obtained through this technique were confirmed by direct regenotyping sequence of PCR products.

### Statistical analysis

Hardy-Weinberg equilibrium was tested using the Hardy-Weinberg package[22]. For genotypic and allelic frequencies, Chi-square test and Spearman’s correlation test were used (SPSS v. 18, SPSS Inc, Chicago, IL, USA). Results were given as mean values, standard deviation and minimum and maximum scores, and OR and 95%CI were also calculated. In each test, a *P* value of less than 0.05 was considered statistically significant.

## RESULTS

The demographic and clinical data of the RA and CD patients are shown in Table 1. Of the 120 RA patients tested for anti-CCP antibodies, 45.8% were found to be positive and 54.2% were negative. Also, 32.5% of RA patients were positive and 67.5% were negative for RF. When the RA patients were classified according to the autoantibody status, no meaningful differences were observed in genotype distributions (*P*=1, OR=0.98, 95%CI=0.39–2.48 and *P*=0.25, OR=0.5, 95%CI=0.19–1.34) in positive compared with negative patients for anti-CCP antibodies and RF, respectively (Table 2).

**Table 1:** Demographic and clinical characteristics of the RA and CD patients

Characteristics		RA patients (n=120)	CD patients (n=52)	Control subjects (n=120)
Demographic				
Age (years)		44.8±11.8	31.69 ±10.9	43.71±9.7
Sex (females/males)		96/24(80/20%)	36/16(69.2/30.7%)	97/23(80.8/19.2%)
Clinical				
Disease duration (years)		7.15± 6.23	2.3±2.48	
Anti-CCP Ab positive*		55/120 (45.8%)	----	----
Anti-CCP Ab negative**		65/120 (54.2%)	----	----
RF positive#		39/120 (32.5%)	----	----
RF negative##		81/120(67.5%)	----	----

Data were presented as the mean±standard deviation or number ratio (percentage).

* no. anti-CCP Ab positive/total no. of patients;

** no. anti-CCP Ab negative/total no. of patients;

# no. RF positive/total no. of patients;

## no. RF negative/total no. of patients; RA, rheumatoid arthritis; CD, celiac disease; Anti-CCP, anti-cyclic citrullinated peptide antibodies; Ab, antibody; RF, rheumatoid factor

**Table 2:** Comparison of *PTPN22* 1858 genotypes in RA patients stratified according to anti-CCP and RF positive with anti-CCP and rheumatoid factor negative

Auto-antibody	CT+TT genotypes n (%)	CC genotype n (%)	T Allele (%)	*x*^2^	*P* value	OR	95%CI
Anti-CCP antibody-negative	10(18.2)	45(81.8)	9.1	0.001	1	0.98	0.39-2.48
	12(18.5)	53(81.5)	9.2				
RF antibody-positive	7(18.0)	32(82.0)	8.9	1.340	0.25	0.50	0.19-1.34
RF antibody-negative	14(17.3)	67(82.7)	8.6				

Referred as subgroups of RA patients; OR, odds ratio; CI, confidence interval.

### Genotype and allele frequencies of the +1858C/T PTPN22 gene polymorphism in rheumatoid arthritis and celiac disease patients

The genotypic and allelic frequencies of the +1858C/T *PTPN22* gene SNP in RA, celiac patients and controls are shown in Table 3. Genotype frequencies for the *PTPN22* 1858T SNP were in Hardy-Weinberg equilibrium in both patients and control cohorts. All genotypes containing the rare 1858T allele were found at increased frequencies in both RA and CD patients. The CT and TT genotypes were present in 18.3% of RA patients and in 26.9% of CD patients but 8.3% in the control group. In addition, the CC genotype significantly declined in RA and CD patients (81.7% and 73.1% as opposed to 91.6%, respectively [χ^2^=6.03, *P*=0.04, OR=2.25, 95%CI = 1.002–5.03 for RA and χ^2^=11.3, *P*=0.004, OR=3.76, 95%CI=1.53–9.29 for CD]). Further, the 1858T allele of *PTPN22* was significantly increased in RA and CD patients compared with the control group subjects (10.0% and 14.4% as opposed to 4.2%, χ^2^=5.35, *P*=0.021, OR=2.56, 95%CI=1.19–5.47 for RA and χ^2^=9.86, *P*=0.002, OR=3.87, 95%CI=1.68–8.95 for CD) (Table 3).

**Table 3:** Distribution of the +1858C/T *PTPN22* gene polymorphism frequency in RA (A) and CD (B) patients and controls

	CS (n=120)		RA patients (n=120)						CD patients (n = 52)					
		
	%	n	%	n	*x*^2^	*P* value	OR	95% CI	%	n	*x*^2^	*P* value	OR	95% CI
Genotype														
CC	91.6	110	81.7	98					73.1	38				
CT	8.4	10	16.7	20	6.03	0.049	2.25	1.002-5.03	25	13	11.3	0.004	3.76	1.53-9.29
TT	0.0	0.0	1.6	2					1.9	1				
Allele														
C	95.8	230	90	216					85.6	89				
T	4.2	10	10	24	5.35	0.021	2.56	1.19-5.47	14.4	15	9.86	0.002	3.87	1.68-8.95

The values were presented as frequency in percentage and number of genotypes or alleles. The frequencies comparison between the groups was analyzed by Chi-Square test. Statistical significance was at *P*<0.05. RA, rheumatoid arthritis; CS, control subjects; OR, odds ratio; CI, confidence interval; CD, celiac disease

## DISCUSSION

The *PTPN22* gene encoding the lymphoid protein tyrosine phosphatase has recently been characterized as a negative regulator of the T-cell and B-cell receptor signaling pathways[15]. There is a dearth of biological data about this protein but a variant allele in this gene conferring an R620W change has been indicated to associate with RA and other autoimmune disease conditions[15,23].

The association between the +1858C/T *PTPN22* SNP and RA has already been documented in several studies[23-28]. Various studies have demonstrated that allelic heterogeneity distribution has an increasing north-south gradient in the frequencies of the 1858T alleles in different European populations[23-25].

In the present investigation, we examined the possible association between *PTPN22* rs2476601 polymorphisms and the risk of RA and CD in a sample of Iranian population. In agreement with our findings, numerous studies have revealed the existence of a positive association between *PTPN22* R620W gene polymorphism and RA diseases in Mexican[^2^], German[8], Swedish[23], Dutch[24], Spanish[25], British[25] and French[25] populations. In contrast, some others have failed to detect any correlation between *PTPN22* 1858 C/T polymorphism and RA risk[16,17,26].

In the present study, the +1858T risk allele was substantially more frequent in RA patients than controls (0.1 vs. 0.042, respectively). This result confirms that +1858T allele behaves as a dominant variant, conferring increased risk of disease. In line with our finding, Hashemi *et al*.[27] have reported that +1858T allele is significantly more frequent in RA patients compared with controls (0.075 vs. 0.017, respectively). Furthermore, Torres-Carrillo *et al*.[^2^] have found the minor allele figures in RA and the control group (0.06 and 0.02, respectively)[^2^].

The association between the minor *PTPN22* allele and autoantibody-positive RA remains controversial. A number of studies have reported an association between the *PTPN22* minor allele and RF-positive disease; however, some other studies have indicated that the minor allele is related to both RF-positive and RF-negative disease[14,23,24]. In our study, no association was observed between the 1858T risk allele and RF-positive disease. Moreover, the comparison of RF-positive and RF-negative genotype frequencies revealed no significant differences between the two groups with *P*=0.25, OR=0.5, and 95%CI=0.19-1.34. In line with our findings, Orozco *et al*.[25] also found no significant difference in the percentage of cases with RF in the TT and CT cases compared with the CC cases. Altogether, our data support the already reported association between the +1858T risk allele and RA in various ethnic populations. Moreover, the role of the *PTPN22* gene, as a susceptibility genetic marker for RA, has been confirmed and highlighted in our study. The association between the +1858C/T *PTPN22* SNP and CD has already been documented in a number of studies[17,18,28]; however, this is the first study that analyzing the relationship between the +1858C/T *PTPN22* SNP and CD in a sample population from Iran. We demonstrated a strong association between the +1858C/T *PTPN22* SNP and CD. In addition, the +1858T allele was noticeably more frequent in CD patients compared with the controls (0.144 as opposed to 0.042, respectively).

*In coincidence* with our study, Santin *et al*.[18] found that the genotypic frequency in carriers of the minor allele (W620 or T allele) among CD patients (18.3%) was substantially higher than healthy controls (11.2%). On the other hand, Zhernakova *et al*.[28] described a tendency of increased frequency of the W620 variant just among early-onset CD patients.

The values were presented as frequency in percentage and number of genotypes or alleles. The frequencies comparison between the groups was analyzed by Chi-Square test. Statistical significance was at *P*<0.05. RA, rheumatoid arthritis; CS, control subjects; OR, odds ratio; CI, confidence interval; CD, celiac disease

The sample size of CD patients in the present study was not very large since the patients were screened and selected from the Iranian Society of Celiac Disease in Khuzestan Province, and members of this community were small in number.

Overall, the data from the present study support the formerly reported association between the +1858T risk allele and CD in a Spanish population[18]. Our findings suggest that the *PTPN22* 1858 genetic variant seems to play an important role in the predisposition of people to CD. The findings of this study together with those of other studies[^2^,^13^,^28^] propose that the +1858C/T *PTPN22* SNP predisposes individuals to autoimmune diseases because of enhanced suppression of T-cell receptor signaling during thymic development, which allows the survival of auto-reactive T-cells. This deficiency in immunological tolerance explains the significance of *PTPN22* in the development of autoimmune diseases.

In conclusion, this study reveals that the +1858T allele in the *PTPN22* gene is associated with RA and CD in a population from the south-west of Iran. Moreover, our findings add to the existing evidence that *PTPN22* is an important genetic risk factor for different autoimmune diseases.
